# Determining the functional role of the *Gluconobacter oxydans* GOX1969 protein as a BamB homolog

**DOI:** 10.1128/spectrum.01060-24

**Published:** 2024-06-25

**Authors:** Ky Ariano, Paul Schweiger

**Affiliations:** 1 Department of Microbiology, University of Wisconsin–La Crosse, La Crosse, Wisconsin, USA; Forschungszentrum Jülich GmbH, lich GmbH, Juelich, Germany

**Keywords:** acetic acid bacteria, β-barrel assembly, BAM complex, *bamB*, PQQ dependent, outer membrane protein

## Abstract

Acetic acid bacteria are used in many industrial processes such as the production of vinegar, vitamin C, the antidiabetic drug miglitol, and various artificial flavorings. These industrially important reactions are primarily carried out by an arsenal of periplasmic-facing membrane-bound dehydrogenases that incompletely oxidize their substrates and shuttle electrons directly into the respiratory chain. Among these dehydrogenases, GOX*1969* in *Gluconobacter oxydans* was predicted to be a pyrroloquinoline quinone-dependent dehydrogenase of unknown function. However, after multiple analysis by a number of labs, no dehydrogenase activity has been detected. Reanalysis of GOX1969 sequence and structure reveals similarities to *Escherichia coli* BamB, which functions as a subunit of the β-barrel assembly machinery complex that is responsible for the assembly of β-barrel outer membrane proteins in Gram-negative bacteria. To test if the physiological function of GOX1969 is similar to BamB in *E. coli*, we introduced the *gox1969* gene into an *E. coli* ∆*bamB* mutant. Growth deficiencies in the ∆*bamB* mutant were restored when *gox1969* was expressed on the plasmid pGox1969. Furthermore, increased membrane permeability conferred by *bamB* deletion was restored upon *gox1969* expression, which suggests a direct link between GOX1969 and a role in maintaining outer membrane stability. Together, this evidence strongly suggests that GOX1969 is functionally acting as a BamB in *G. oxydans*. As such, functional information on uncharacterized genes will provide new insights that will allow for more accurate modeling of acetic acid bacterial metabolism and further efforts to design rational strains for industrial use.

**IMPORTANCE:**

*Gluconobacter oxydans* is an industrially important member of the acetic acid bacteria. Experimental characterization of putative genes is necessary to identify targets for further engineering of rational acetic acid bacteria strains that can be used in the production of vitamin C, antidiabetic compounds, artificial flavorings, or novel compounds. In this study, we have identified an undefined dehydrogenase GOX1969 with no known substrate and defined structural similarities to outer membrane biogenesis protein BamB in *E. coli* K12. Furthermore, we demonstrate that GOX1969 is capable of complementing *bamB* knockout phenotypes in *E. coli* K12. Taken together, these findings enhance our understanding of *G. oxydans* physiology and expand the list of potential targets for future industrial strain design.

## INTRODUCTION

Acetic acid bacteria are a group of industrially relevant Gram-negative aerobes that are commonly exploited for their ability to oxidize a wide variety of growth substrates (1–3). One member of this group, *Gluconobacter oxydans*, is of interest as its unique metabolism makes it a preferential candidate for large-scale production of numerous products of chemical, cosmetic, and pharmaceutical importance (4, 5). *G. oxydans* lacks multiple enzymes (phosphofructokinase, phosphoenolpyruvate synthase, succinate dehydrogenase, and succinyl coenzyme A synthetase) present in traditional central carbon metabolism pathways and subsequently overcomes these metabolic shortcomings with a wide array of periplasmic-facing dehydrogenases (1–3, 6, 7). These dehydrogenases incompletely oxidize a broad range of substrates and release their products directly into the periplasm, where they are free to diffuse into the media through β-barrel outer membrane porins. The periplasmic localization of these dehydrogenases contributes to the industrial viability of *G. oxydans* as the resulting metabolic byproducts can be harvested without the lysis of the bacterial population. The discovery, characterization, and genetic manipulation of *G. oxydans* is of interest for improving the synthesis of metabolic products such as vitamin C, the antidiabetic drug miglitol, erythrose, and artificial flavorings (4). The overexpression of various dehydrogenases has been explored for increasing growth rates and product yields with great success (8–11). As such, defining the roles of uncharacterized genes broadens our understanding of acetic acid bacteria physiology and allows further refinement of *G. oxydans* metabolism within an industrial context.

*G. oxydans* GOX1969 was originally annotated as a pyrroloquinoline quinone (PQQ)-dependent dehydrogenase of unknown function (1). Despite this annotation, no dehydrogenase activity has been detected upon experimental analysis of GOX1969 (7). Reanalysis of GOX1969 suggests alternative likeness to the BamB subunit of the β-barrel assembly machinery (BAM) complex (7). BamB belongs to the PQQ-dependent dehydrogenase-like family, although known BamB proteins do not require the PQQ cofactor. The BAM complex is composed of five subunits (BamA–BamE) which orchestrate the biogenesis and folding of numerous β-barrel outer membrane proteins (OMPs) (12–14). The BamB subunit principally handles the binding and passage of unfolded OMP precursors from periplasmic chaperones to the transmembrane BamA subunit of the BAM complex (15, 16). Many of the outer membrane proteins folded by the BAM complex function as porins for the passage of nutrients and metabolites across the outer membrane (17). As such, the BAM complex is likely crucial for *G. oxydans* to prevent the accumulation of toxic metabolic byproducts within the periplasmic space.

To see if GOX1969 shares physiological function with *Escherichia coli* BamB, we utilized sequence analysis and predictive modeling of GOX1969 to identify conserved structural features that are shared across functional BamB proteins. Conserved structural characteristics within GOX1969 suggest the presence of a BamA binding interface, docking sites for SurA, and unfolded OMP handling residues. Furthermore, we investigated if *gox1969* expression on the inducible plasmid pGox1969 could restore phenotypic differences imposed by *bamB* deletion in *E. coli* K12. Expression of *gox1969* was capable of restoring growth defects and recovering increased membrane permeability in the *E. coli* K12 ∆*bamB* mutant. Together, these data strongly suggest functional similarity between *G. oxydans* GOX1969 and *E. coli* BamB.

## RESULTS

### Structural prediction and identification of functional residues within GOX1969

**Fig 1:**
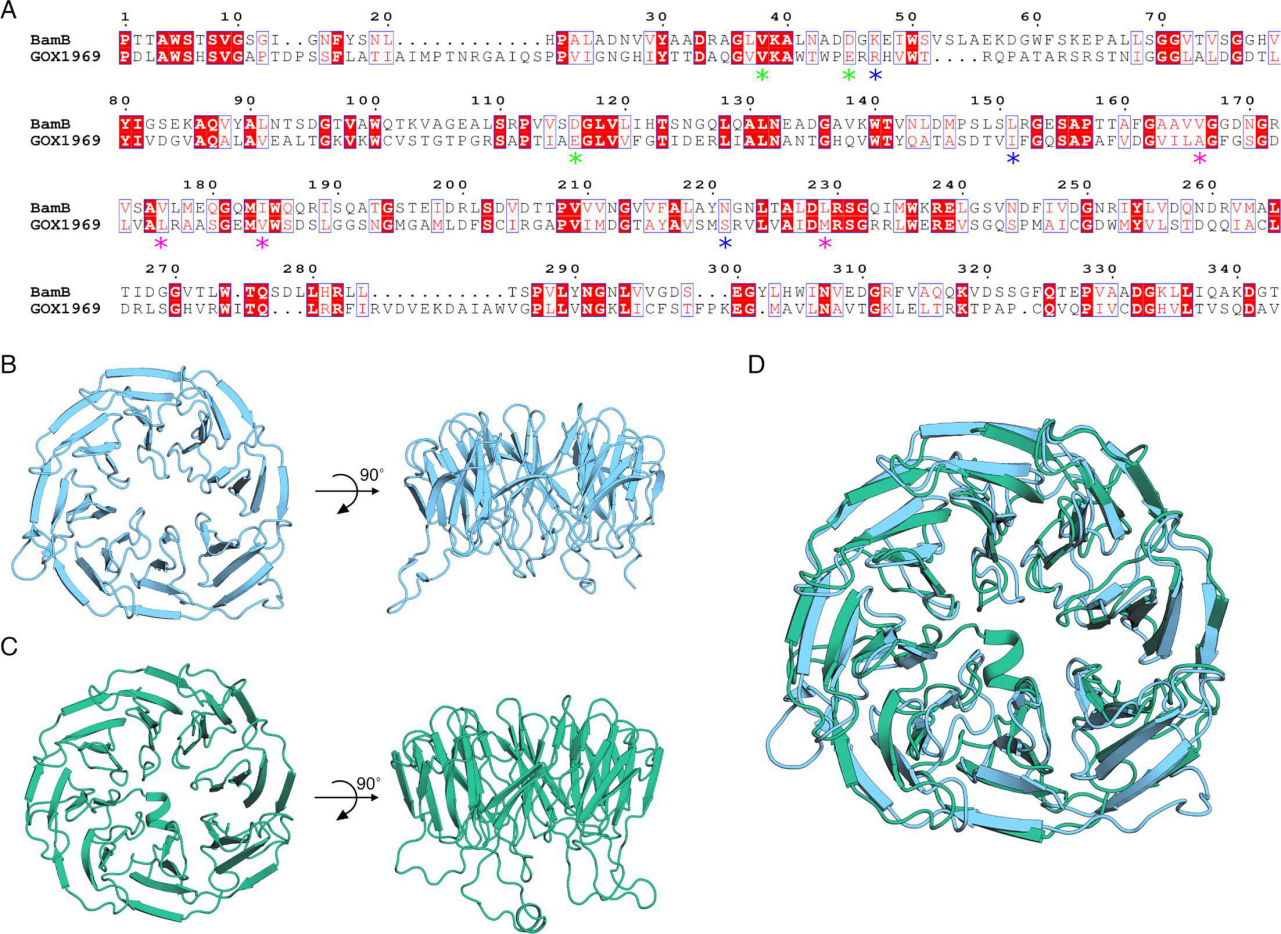
Sequence and structural alignment of BamB with GOX1969 highlights similarities. (**A**) Sequence alignment of *G. oxydans* GOX1969 (UniProt: Q5FPJ1) and *E. coli* BamB (UniProt: P77774) using Smith-Waterman local alignment. Sequences share 24.40% identity and 43.97% similarity. Displayed sequences are trimmed to depict the region of highest alignment that contains predicted important residues. GOX1969 amino acids 103–465 and BamB amino acids 44–386 are shown with numbering starting at the beginning of the trimmed sequences. Red boxes indicate strict sequence identity, and red letters indicate sequence similarity. Blue frames indicate regions of similarity between the groups. Residues involved in BAM complex interactions are denoted with green stars; residues involved in SurA binding are denoted with blue stars; and residues involved in OMP substrate handling are denoted with pink stars. Alignment annotations were produced using ESPript (v.3.0) (20). (**B**) Top and side views of *E. coli* BamB (PDB: 3P1L). (**C**) Top and side views of GOX1969 structure as predicted by AlphaFold2. (**D**) Alignment of *E. coli* BamB crystal structure (PDB: 3P1L, shown in blue) and AlphaFold2 predicted *G. oxydans* GOX1969 structure (shown in green).

The sequence similarity of GOX1969 and BamB was previously noted (7). Alignment of GOX1969 and BamB showed sequence similarity of 43.97% and sequence identity of 24.40% (Fig. 1A). Furthermore, alignment of GOX1969 with eight annotated BamB proteins among related acetic acid bacteria showed a high degree of sequence similarity between each sequence (Fig. S1). To explore the structural similarities between *G. oxydans* GOX1969 and *E. coli* BamB, predictive modeling of GOX1969 using AlphaFold2 was done (18). The predicted model of GOX1969 has high overall perresidue confidence scores with exceptions being observed on N-terminal residues 1–62 and on several loops that extend away from the main body of the model (Fig. S2A). To refine our model, the GOX1969 sequence was analyzed with SignalP (v.6.0) (19), which revealed the presence of a TAT signal peptide with a cleavage site after G37 (Fig. S2B). Furthermore, we identified an N-terminal peptide that is absent in BamB (Fig. S2C). This loop is composed of residues 38–105 and was removed along with the signal peptide on the GOX1969 model for visual clarity.

*E. coli* BamB has an eight bladed β-propeller confirmation with each blade being radially arranged around a central pore. Each blade consists of four antiparallel β-sheets that are linked together through connecting loops that vary between 2 and 20 amino acids in length (Fig. 1B). The predicted model of *G. oxydans* GOX1969 shows a similar β-propeller organization with each blade also being composed of four antiparallel β-sheets with connecting loops of 2–25 amino acids in length (Fig. 1C). GOX1969 also shows radial arrangement of its β-blades; however, the presence of an unstructured N-terminal 106 amino acid tail obstructs the pore in the predicted model (Fig. S2C). Alignment of the two structures highlights their high degree of structural similarity despite sharing only 24.40% sequence identity (Fig. 1D).

**Fig 2:**
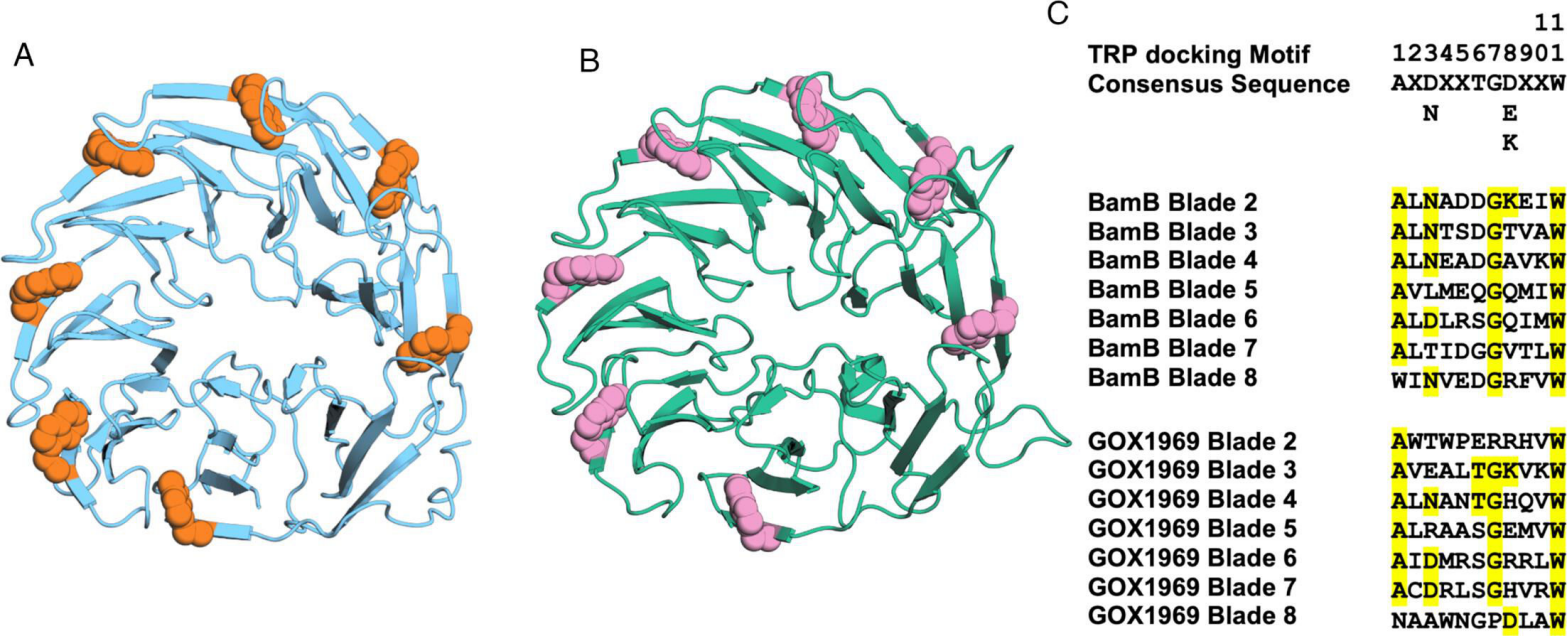
GOX1969 possesses tryptophan docking motifs. (**A**) Tryptophan docking residues W93, W143, W183, W228, W279, W317, and W348 displayed on *E. coli* BamB crystal structure (PDB: 3P1L) in orange spheres. W93 is on the bottom of the structure, and each residue is displayed sequentially in clockwise order. (**B**) Tryptophan docking residues W107, W166, W212, W252, W297, W348, and W386 displayed on AlphaFold2 predicted *G. oxydans* GOX1969 structure in pink spheres. W107 is on the bottom of the structure, and each residue is displayed sequentially in clockwise order. (**C**) Tryptophan docking motif sequences of *E. coli* BamB and *G. oxydans* GOX1969 aligned with the tryptophan docking motif consensus sequence.

GOX1969 shares various characterized structural features with *E. coli* BamB, including the presence of many important functional residues. These residues play a role in unfolded OMP peptide receiving and handling. Seven tryptophan docking motifs are medially located on the outermost β-sheet on blades 2–8 in *E. coli* BamB with each roughly following the tryptophan docking motif consensus sequence [AXX(D/N)XXTG(D/E/K)XXW] (21). These motifs are expected to form an inter-blade tryptophan stabilizing girdle that is integral to β-propeller protein stability (22). GOX1969 sequence and structure displays seven tryptophan docking motifs that are similarly located on the outermost β-sheet on blades 2–8 (Fig. 2). Interestingly, both proteins lack the starting alanine residue of the tryptophan docking motif consensus sequence on the eight blade. Between BamB and GOX1969, there are no instances of a docking motif that fully conforms to the tryptophan docking consensus sequence. However, the positioning of each tryptophan side chain against the backbone of the adjacent blade highly suggests that this stabilizing girdle is present in both proteins.

**Fig 3:**
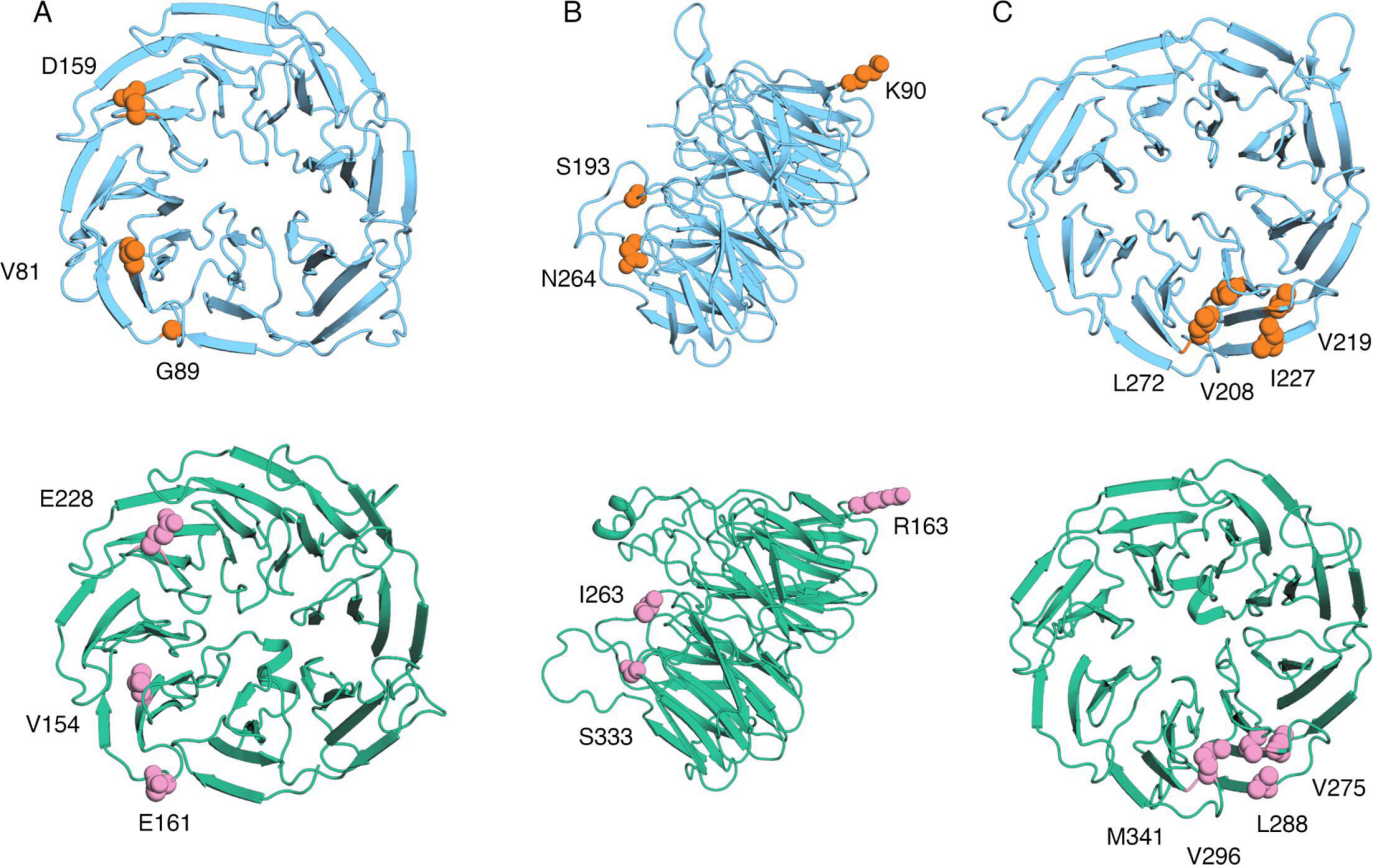
Functional residues defined in *E. coli* BamB are found in GOX1969 at similar loci. (**A**) Residue side chains involved in stabilizing BamA-SurA docking displayed on *E. coli* BamB crystal structure (PDB: 3P1L, shown in blue) in orange spheres (top). Residue side chains that align with *E. coli* BamB SurA docking active sites shown on AlphaFold2 predicted *G. oxydans* GOX1969 structure (shown in green) in pink spheres (bottom). (**B**) Residue side chains that form the BamA-BamB interface displayed on *E. coli* BamB crystal structure (PDB: 3P1L, shown in blue) in orange spheres (top). Residue side chains that align with *E. coli* BamB BAM complex interface shown on AlphaFold2 predicted *G. oxydans* GOX1969 structure (shown in green) in pink spheres (bottom). (**C**) Residue side chains involved in OMP substrate displayed on *E. coli* BamB crystal structure (PDB: 3P1L, shown in blue) in orange spheres (top). Residue side chains that align with OMP binding sites shown on AlphaFold2 predicted *G. oxydans* GOX1969 structure (shown in green) in pink spheres (bottom).

BamB stabilizes the interaction between incoming periplasmic chaperones facilitating efficient transfer and delivery of OMP substrates to the BAM complex (15, 23–25). Sequence alignment of *E. coli* BamB with GOX1969 indicates the presence of multiple residues that are involved in BamA-BamB interactions, SurA binding, and OMP substrate handling within both sequences (Fig. 1A). Comparing the location of these interacting residues on the three-dimensional (3D) models shows that GOX1969 residues of interest are located in very similar positions when compared to *E. coli* BamB. GOX1969 possesses three congruent residues with BamB that are involved in binding SurA (*gox0301* in *G. oxydans*) (25). BamB residues D159, V81, and G89 show similar location and alignment with GOX1969 E228, V154, and E161, respectively (Fig. 3A). BamB residues S193, N264, and K90 are involved in complex formation between the central BAM complex subunit BamA (*gox1818* in *G. oxydans*) (14, 15). GOX1969 residues I263, S333, and R163 align with corresponding residues in BamB and are located similarly in their respective 3D structures (Fig. 3B). Furthermore, BamB residues L272, V208, I227, and V219 form a hydrophobic binding pocket that interacts with unfolded OMP precursors (15). GOX1969 also exhibits the presence of a hydrophobic binding pocket composed of residues M341, V296, L228, and V275 that share alignment and location with their BamB counterparts (Fig. 3C). All of the above residues present in GOX1969 show spatial similarities with their counterparts on BamB (Fig. 3).

### Gox1969 expression recovers growth defects in an *E. coli* K12 ∆*bamB* mutant

BAM complex deletion mutants exhibit growth defects and increased membrane instability in *E. coli* (26). Efforts to create a *G. oxydans* 621H ∆*gox1969* mutant using a markerless deletion strategy were previously unsuccessful (7). Transposon-based Knockout Sudoku also failed to disrupt the *gox1969* homolog in *G. oxydans* strain NRRL B-58 (5). It is possible that the inability to create a knockout is due to polar effects on the downstream Der GTPase that is essential in many bacteria due to its role in ribosome biosynthesis (27). Yet *bamB* is nonessential in *E. coli* and has been successfully deleted (28, 29). This suggests that *gox1969* could be essential in *G. oxydans*. As such, we sought to complement *bamB* with *gox1969* in an *E. coli* K12 ∆*bamB* background. The *gox1969* gene was amplified from wild-type *G. oxydans* 621H and cloned into pASK-IBA3 to produce the pGox1969 plasmid. *E. coli* ∆*bamB* was transformed with pGOX1969 to produce *E. coli* ∆*bamB* pGox1969. Empty vector pASK-IBA3 was transformed into wild-type *E. coli* K12 and *E. coli* ∆*bamB* to control for metabolic burden imposed by plasmid maintenance.

**Fig 4:**
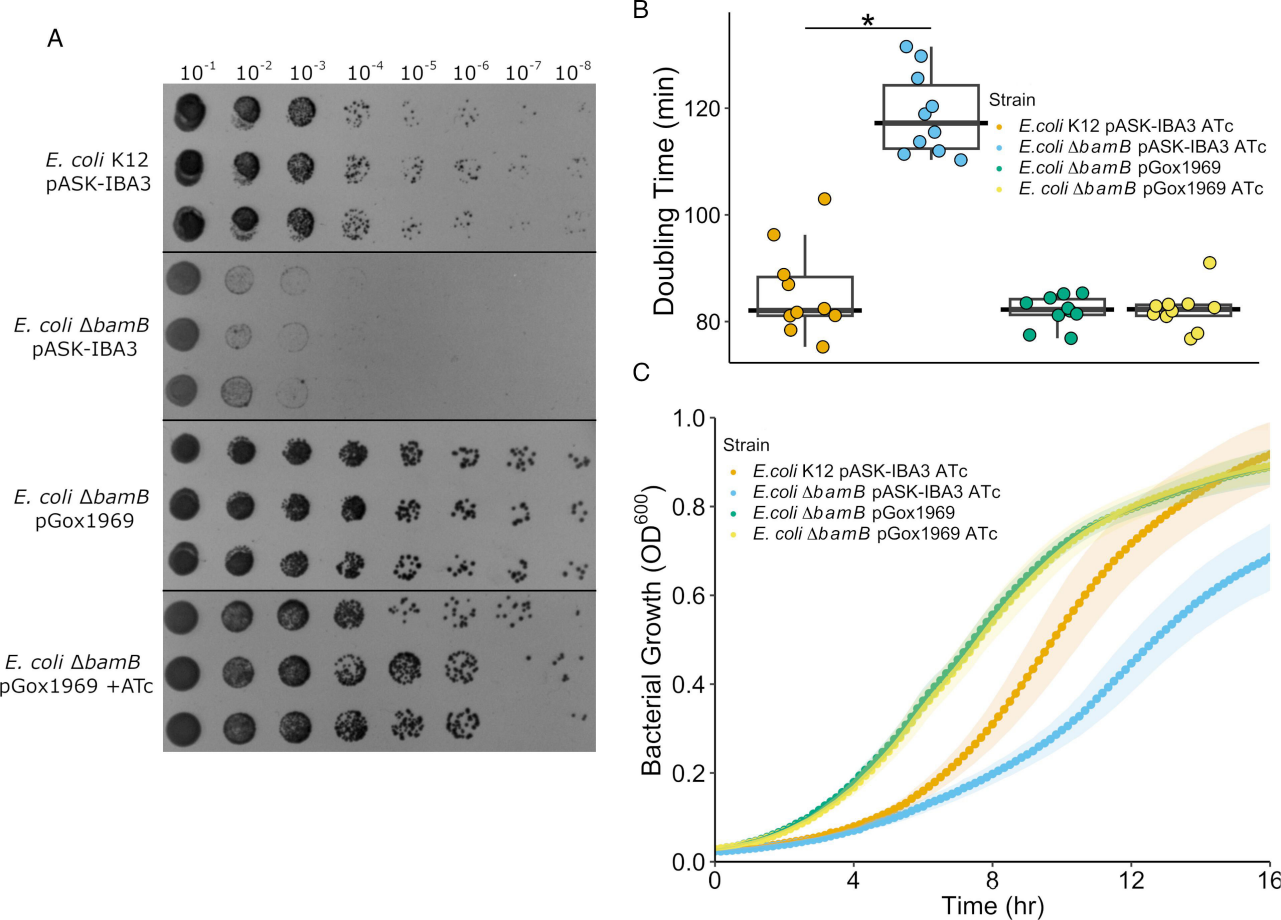
Induction of *gox1969* recovers growth defects in an *E. coli* ∆*bamB* mutant. (**A**) Serial dilution spot plates of *E. coli* K12 strains grown on lysogeny broth (LB) agar at 24°C. Cells were harvested in log phase growth, normalized to a starting OD_600_ of 0.05 in LB, and diluted tenfold in a 96 well plate. Each dilution was spotted onto LB agar in triplicate (*n* = 3). Images were taken after 72 h of growth. (**B**) Average doubling times of *E. coli* K12 strains grown in LB at 24°C with 250-rpm shaking. Wild type *E. coli* K12 and *E. coli* ∆*bamB* contained empty vector pASK-IBA3. Doubling times were calculated using the Growthcurver R package (32). An asterisk (*) indicates a *P* < 0.05. (**C**) Growth curves of *E. coli* K12 mutants grown in LB at 24°C with 250-rpm shaking. Wild type *E. coli* K12 and *E. coli* ∆*bamB* contained empty vector pASK-IBA3. Cells were harvested in log phase, normalized to a starting OD_600_ of 0.05 in LB, and distributed onto a 96 well plate (*n* = 10). 95% confidence intervals are plotted as ribbons.

Serial dilution spot plates were done to qualitatively analyze if expression of *gox1969* could recover growth defects imposed by *bamB* deletion in *E. coli* K12 as it was previously shown that *E. coli* with mutated BAM complex components grow poorly at reduced temperatures (13, 29–31). Wild-type *E. coli* K12 pASK-IBA3 had visible growth down to the 10^−8^ dilution spot. The *E. coli* ∆*bamB* pASK-IBA3 mutant had deficient growth when compared to *E. coli* K12 pASK-IBA3 with little growth being observed below the 10^−3^ dilution spots (Fig. 4A). In contrast, the *E. coli* ∆*bamB* strain harboring the pGox1969 plasmid recovered the growth deficiencies observed in the *bamB* deletion mutant. Regardless of induction, *E. coli* ∆*bamB* pGox1969 displayed growth patterns nearly identical to wild-type *E. coli* K12 pASK-IBA3 with observable growth being present in the 10^−8^ dilution spots (Fig. 4A).

Growth curves were produced, and doubling times were calculated to quantify the observed *bamB* knockout mutant growth phenotype and apparent *gox1969* restoration of these defects. Consistent with the spot plate results, the *E. coli* ∆*bamB* pASK-IBA3 mutant had significantly slower doubling times in comparison to wild-type *E. coli* K12 pASK-IBA3 (*P* < 0.0001). Average doubling times for wild-type *E. coli* K12 pASK-IBA3 were 85.5 minutes, while the *bamB* deletion mutant doubled at an average of 118.9 minutes (Fig. 4B). Complementation of *bamB* with pGox1969 restored doubling times to that of the wild-type strain regardless of induction. *E. coli* ∆*bamB* pGox1969 doubling times with and without Anhydrotetracycline (ATc) induction were 81.9 and 82.1 minutes, respectively (Fig. 4B).

Growth curves of the mutants yielded results similar to the spot plates, with the ∆*bamB* mutant exhibiting reduced growth phenotypes (Fig. 4C). When compared to the wild type, the ∆*bamB* mutant failed to reach comparable cell densities and had an extended lag phase. Final optical density for *E. coli* ∆*bamB* pASK-IBA3 averaged 0.7 OD_600_, whereas wild-type *E. coli* K12 pASK-IBA3 reached an average optical density of 0.9 OD_600_. Expression of *gox1969* in the ∆*bamB* strain appears to recover these growth deficiencies. Regardless of induction, the *E. coli* ∆*bamB* pGox1969 mutant exhibits a shorter lag phase in comparison to *E. coli* ∆*bamB* pASK-IBA3 and *E. coli* pASK-IBA3. Furthermore, *E. coli* ∆*bamB* pGox1969 reaches final cell densities that are comparable to the wild-type strain with both induced and uninduced samples reaching average optical densities of 0.9 OD_600_.

Leaky induction of the Tet promoter could explain the lack of growth differences between induced and uninduced *E. coli* ∆*bamB* pGox1969 samples. Reverse transcription quantitative PCR (RT-qPCR) of the *gox1969* transcript was done to confirm the efficacy of *gox1969* induction by ATc. The transcript abundance of *gox1969* increased by approximately 130-fold when induced. RT-qPCR of the *bamB* transcript was done with wild-type *E. coli* K12 to determine the normal background level of *bamB* expression. Despite increasing reaction template from 0.5 to 18.0 ng/µL, *bamB* transcript remained below the detectable limit. In contrast, *gox1969* expression was detectable in uninduced *E. coli* ∆*bamB* pGox1969 even when using 0.5 ng/µL of template, suggesting a low level of leaky expression. Taken together, these data imply that only low levels of BamB are necessary to confer function. As such, low levels of leaky *gox1969* expression when ATc is absent is sufficient for complementation of ∆*bamB* phenotypes.

### *Gox1969* expression restores membrane integrity in *E. coli* ∆*bamB* cells

**Fig 5:**
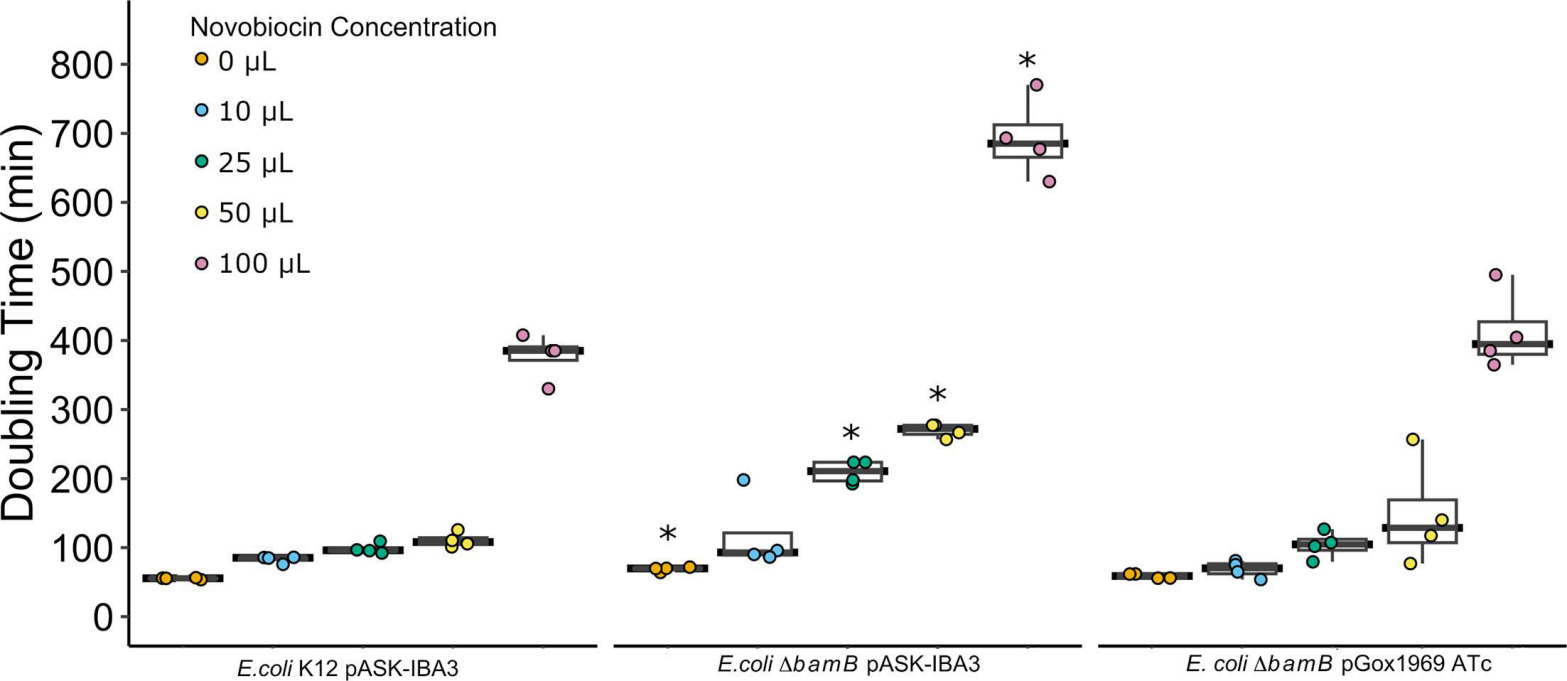
Expression of *gox1969* restores innate resistance to DNA gyrase inhibitor novobiocin in *E. coli* ∆*bamB* strain. Average doubling times of *E. coli* K12 strains grown in LB supplemented with various concentrations of novobiocin at 24°C with 250-rpm shaking. Wild-type *E. coli* K12 and *E. coli* ∆*bamB* contained empty vector pASK-IBA3. Cells were harvested in log phase, normalized to a starting OD_600_ of 0.1 in LB, and distributed onto a 96-well plate (*n* = 4). Doubling times were calculated from growth curves using the Growthcurver R package (32). An asterisk (*) indicates a *P* value of <0.05.

Deletion of accessory OMP biogenesis proteins is lethal or hinders OMP folding efficiency, leading to the destabilization of the outer membrane (23, 30). Deletion of *bamB* is known to increase membrane permeability to hydrophobic antibiotics (23, 33). Consequently, cells were subjected to growth in media supplemented with increasing concentrations of the antibiotic novobiocin to characterize the impact that *bamB* deletion has on membrane stability. Novobiocin is a bulky DNA gyrase inhibitor that has difficulty crossing healthy Gram-negative outer membranes due to lipopolysaccharides acting as a permeability barrier (33, 34). Wild-type *E. coli* pASK-IBA3 growth was uninhibited in media with 10-, 25-, and 50-µM novobiocin. However, a significant increase in *E. coli* K12 pASK-IBA3 doubling time was observed in lysogeny broth (LB) supplemented with 100-µM novobiocin (*P* < 0.0001). In contrast to the wild-type *E. coli* K12 pASK-IBA3 novobiocin sensitivity profile, the *E. coli* ∆*bamB* pASK-IBA3 mutant was significantly more sensitive to novobiocin (25 µM, *P* < 0.0001; 50 µM, *P* < 0.0001, and 100 µM, *P* < 0.0001). Large increases in doubling time were observed in media containing 25-, 50-, and 100-µM novobiocin (Fig. 5). To test if the ∆*bamB* novobiocin sensitivity phenotype could be restored through complementation with *gox1969*, *E. coli* ∆*bamB* pGox1969 cells were grown in the presence of novobiocin. Expression of *gox1969* in *E. coli* ∆*bamB* restored the observed novobiocin sensitivity and restored doubling times to significantly similar levels to that of wild-type *E. coli* K12 pASK-IBA3 (Fig. 5) (0 µM, *P* = 0.063; 10 µM, *P* = 0.080; 25 µM, *P* = 0.259; 50 µM, *P* = 0.210; and 100 µM, *P* = 0.139).

## DISCUSSION

Previously annotated as an uncharacterized PQQ-containing oxidoreductase, attempts have been made to characterize GOX1969 dehydrogenase activity without success (7). GOX1969 shares numerous similarities to the BamB subunit of the BAM complex, which leads us to believe that GOX1969 possesses alternative functional capabilities. The BamB protein is present as a BAM complex subunit across numerous proteobacterial lineages (34). Furthermore, deletion of *bamB* genes causes altered phenotypes in outer membrane permeability, OMP biogenesis, and virulence capabilities (26, 35, 36). In this study, we examined the structural characteristics of GOX1969 in comparison to *E. coli* BamB and its ability to fully complement ∆*bamB* phenotypes in *E. coli* K12 to better clarify the role of GOX1969 in *G. oxydans* physiology.

*E. coli* BamB and its related homologs take on an eight-bladed β-propeller confirmation with radial symmetry around a central pore. Additionally, the presence of tryptophan docking motifs is a consistent feature among these proteins (22, 24, 34). Predictive modeling reveals GOX1969 to have a β-propeller structure composed of eight β-blades with tryptophan docking motifs located on the outermost β-strand (Fig. 2B and C). Comparison between GOX1969 and *E. coli* BamB illustrates their similar cylindrical shape and β-blade composition, which suggests a high degree of functional similarity. One notable difference is that the central pore of GOX1969 is obstructed by an N-terminal loop, whereas the *E. coli* BamB pore is open. The openness of this central pore has been suggested to be a differential feature between BamB-like proteins and PQQ-dehydrogenases. However, mutational obstruction of the central pore fails to induce a ∆*bamB* phenotype, and BamB remains functional (22). Although physical openness is not essential to BamB function, the obscuring N-terminal tail may be due to either organismal differences or artifacts introduced during structure prediction.

Regardless of the discrepancies between the *E. coli* BamB crystal structure and the predicted GOX1969 model, the location and alignment of residues involved in maintaining structural stability, BAM complex binding, SurA interactions, and unfolded OMP substrate handling provide much stronger evidence that these two proteins share functional capabilities. The role of BamB in orchestrating substrate delivery is primarily facilitated through stabilizing interactions between central BAM complex subunit BamA and periplasmic chaperone SurA (15, 23, 24, 37). Sequence alignment of *E. coli* BamB with GOX1969 indicates the presence of multiple residues involved in BamA-BamB interactions, OMP substrate binding, and SurA docking within both sequences. We found that the locations of these residues on the 3D structures reside in similar positions. The proximity of these interacting residues in GOX1969 to those found in *E. coli* BamB is highly suggestive that GOX1969 is functionally similar to BamB *in vivo*. We recognize that the limitations of predicted structures as bioinformatic models are not infallible. However, together these data provide a basis for further experimental comparison between BamB and GOX1969 function.

Mutagenesis of BAM complex subunits and OMP periplasmic chaperones induces growth defects at lower temperatures or in the presence of permeabilizing agents such as sodium dodecyl sulfate (13, 29–31). In accordance with the numerous studies that find these growth defects, our *E. coli* ∆*bamB* mutant had an extended lag phase and grew significantly slower when compared to wild-type *E. coli* K12. Additionally, we demonstrated that BamB deletion causes detrimental effects on membrane health as assessed by novobiocin sensitivity (14, 23, 26). Compromised membranes allow for higher intracellular novobiocin concentrations, which presents as increased sensitivity at lower concentrations. As such, we observed the *E. coli* ∆*bamB* mutant to be far more susceptible to novobiocin.

Expression of *gox1969* in the *bamB* knockout mutant restored both the observed growth deficiencies and novobiocin sensitivity phenotype. In both growth analysis and novobiocin sensitivity assays, *E. coli* K12 pASK-IBA3 and *E. coli* ∆*bamB* pGox1969 strains are phenotypically indistinguishable. These data indicate complete complementation of ∆*bamB* phenotypes by *gox1969* expression, which strongly suggests that GOX1969 is not a PQQ-containing dehydrogenase but is instead a functional BamB.

Unexpectedly, we found that the *E. coli* ∆*bamB* pGox1969 strain grew marginally better than wild-type *E. coli* K12, having slightly faster doubling times and reaching optical densities of 0.9 around 30 minutes faster, regardless of induction by ATc. We initially attributed this to improper induction of the pGox1969 plasmid. However, RT-qPCR results indicate an average 130-fold difference in *gox1969* transcript abundance in the presence of ATc. Attempts to quantify normal background *bamB* expression in wild-type *E. coli* K12 were unsuccessful despite increasing RNA concentrations 36-fold. Because of this, we speculate that BamB is normally present in very low amounts and that any leakiness of the *gox1969* expression system produces enough GOX1969 protein to restore growth phenotypes. Furthermore, we suspect that overexpression of *gox1969,* even in low amounts in uninduced cells, resulted in the aforementioned improved growth with slightly shorter lag phases and doubling times in comparison to the wild type. This may be due to the presence of more functional BamB compared to wild-type cells.

Difficulty in generating a *G. oxydans* ∆*gox1969* strain may be attributed to the importance of periplasmic nutrient uptake in acetic acid bacteria. Periplasmic dehydrogenase activity comprises a large part of *G. oxydans* metabolism. Thus, disruption of OMP biogenesis machinery may reduce metabolite translocation to a point of toxicity. Buildup of various intermediate metabolites within the periplasm may also become toxic and serve as one such mechanism for *gox1969* knockout, resulting in a lethal phenotype.

Experimental characterization of GOX1969 provides new physiological roles for similar putative PQQ-containing oxidoreductases with no apparent dehydrogenase functionality. Due to the presence of tryptophan docking motifs being a shared structural feature between PQQ-containing oxidoreductases and BamB proteins, initial incorrect annotation of these genes is understandable, yet further analysis of conserved BamB features may provide an effective route for delineating these two protein types in other acetic acid bacteria (22). Successful complementation of *E. coli ∆bamB* growth and novobiocin tolerance phenotypes by *gox1969* expression strongly points to GOX1969 being the functional BamB subunit of the *G. oxydans* BAM complex. Further investigation of GOX1969 and the BAM complex will provide new insights into acetic acid bacteria physiology and will indicate potential new targets for bioengineering more efficient industrial acetic acid bacterial strains.

## MATERIALS AND METHODS

### Bacterial strains and culture conditions

*E. coli* strains (Table 1) were routinely grown at 37°C or 24°C in LB (5-g/L yeast extract, 10-g/L tryptone, and 10-g/L sodium chloride) with 250-rpm shaking. Ampicillin (100 µg/mL) was added to the media for maintenance of the pGox1969 and pASK-IBA3 plasmids. Kanamycin (50 µg/mL) was added to the media for selection of *E. coli* ∆*bamB* strains. *G. oxydans* 621H was grown in yeast mannitol broth (20-g/L mannitol and 6-g/L yeast extract) at 30°C with 250-rpm shaking. Agar was added to 1.5% when making solid media. *E. coli* strains were transformed by electroporation, and *E. coli* K12 and *E. coli ∆bamB* cells were made electrocompetent using a rapid protocol (38). ATc was added to growth media for the induction of the Tet promoter on pGox1969 to a final concentration of 25 ng/mL.

**TABLE 1:** Bacterial strains, plasmids, and primers used in this study

Strain	Description	Source
*E. coli* K12	Wild type	ATCC 10798
*E. coli* ∆*bamB*	*bamB*760(del)::*kanR*	Yale CGSC JW2496-3
*G. oxydans* 621H	Wild type	DSM 2343
Plasmids		
pASK-IBA3	*ampR*	IBA Lifesciences
pGox1969	*ampR,* pASK-IBA3 with *gox1969* cloned into the upstream BsaI and HindIII sites after streptag; uses RBS from pASK-IBA3 and Tet promoter to induce expression of *gox1969*	This study
Primers		
rrsA_rt_F	CTCTTGCCATCGGATGTGCCCA	(39)
rrsA_rt_R	CCAGTGTGGCTGGTCATCCTCTCA	(39)
gox1969_rt_F	CTTTCCCGATCTTCCCTTTC	This study
gox1969_rt_R	GCTTCTTGTCGTCCTCGAAC	This study
bamB_rt_F	TGCATTACTTTCTGGCGGTG	This study
bamB_rt_R	GATTAACACCAGACCGTCGC	This study
gox1969F	ATGGTAGGTCTCAAATGATGCGCCGTCCTGTTCTTTCC	This study
gox1969R	ATGGTAAAGCTTTCAGCCATAGGCCCGCAGAAC	This study
pASK_F	GAGTTATTTTACCACTCCCT	IBA Lifesciences
pASK_R	CGCAGTAGCGGTAAACG	IBA Lifesciences

### Materials and molecular techniques

Standard molecular techniques were done according to manufacturer or standard protocols (40). The Qiagen DNeasy UltraClean kit was used to purify genomic DNA (Qiagen, Germantown, MD, USA). The GeneJET Plasmid Miniprep Kit (Thermo Fisher Scientific, Waltham, MA, USA) was used to extract plasmid DNA. The SV total RNA isolation system (Promega, Madison, WI, USA) was used to extract bacterial RNA following the protocol for isolation of RNA from Gram-negative bacteria. RNA samples were further purified using the TURBO DNA-*free* kit (Invitrogen by Thermo Fisher Scientific, Carlsbad, CA, USA) and quantified using the QuantiFluor RNA System (Promega). Phusion DNA polymerase and DreamTaq polymerase, FastDigest restriction enzymes, and T4 ligase were purchased from Thermo Fisher Scientific. Eurofins Genomics supplied all primers used for PCR and performed DNA sequencing (Louisville, KY, USA).

### Cloning of *gox1969* into pASK-IBA3

The *gox1969* gene was amplified from *G. oxydans* using gox1969F/gox1969R primers (Table 1) and Phusion DNA polymerase. The *gox1969* amplicon was cut with BsaI/HindIII and ligated with the similarly cut pASK-IBA3 plasmid using T4 ligase to construct the pGox1969 plasmid. The pGox1969 plasmid was transformed into *E. coli* K12 by electroporation and plated on LB_amp_. Positive colonies were confirmed by colony PCR using pASK_F/pASK_R primers (Table 1) and DreamTaq DNA polymerase following the manufacturer’s recommendations. Positive clones were confirmed by DNA sequencing (Eurofins Genomics).

### Sequence alignments and predictive modeling

Sequence were aligned using Smith-Waterman local alignment or T-coffee in SnapGene (v.7.0.2) (41). Alignments were annotated using ESPript (v.3.0) (20). Modeling was carried out using AlphaFold2, and structures were manipulated using PyMOL (18, 42).

### Serial dilution spot plates

Overnight cultures of *E. coli* K12 pASK-IBA3, *E. coli ∆bamB* pASK-IBA3, and *E. coli ∆bamB* pGox1969 were diluted to approximately 0.05 OD_600_, induced by addition of ATc (25 ng/mL), and incubated at 24°C with 250-rpm shaking for 4 h. Cells were diluted 10^−1^ to 10^−8^ with LB in a 96-well plate. Each dilution was spotted (10 µL) onto LB plates containing the appropriate antibiotics. Plates were incubated for 72 h at 24°C prior to imaging using a Gel Doc EZ imager (Bio-Rad Laboratories, Hercules, CA, USA) with Image Lab (v.5.2.1). Plates were exposed for 1 minute and imaged using the “stain-free” setting with high at −3,000 and low at −600.

### Growth curves and doubling time calculations

Overnight cultures of *E. coli* K12 pASK-IBA3, *E. coli* ∆*bamB* pASK-IBA3, and *E. coli* ∆*bamB* pGox1969 were diluted to approximately 0.1 OD_600_, and the pGox1969 plasmid was induced by the addition of ATc to a final concentration of 25 ng/mL. Cultures were grown at 24°C with 250-rpm shaking. At 4 h post induction, cultures were normalized to an OD_600_ of 0.1 in LB; 200 µL of culture was distributed onto a 96-well plate; and growth was monitored using a SpectraMax M3 plate reader (Molecular Devices, San Jose, CA, USA) at 600 nm, 24°C, with shaking in between reads. Growth assays were done with at least three biological replicates each with three technical replicates. Doubling times were calculated and growth curves were constructed in RStudio using the Growthcurver package (32, 43) (Script 1).

### RT-qPCR of *gox1969* and *bamB* transcript

Overnight cultures of *E. coli* K12 pASK-IBA3, *E. coli* ∆*bamB* pASK-IBA3, and *E. coli* ∆*bamB* pGox1969 were diluted to approximately 0.1 OD_600_ and the pGox1969 plasmid was induced by the addition of ATc to a final concentration of 25 ng/mL. Cultures were induced at 24°C with 250-rpm shaking. At 4 h post induction, total cellular RNAs were isolated using the SV total RNA isolation system (Promega), and DNA contamination was removed using the TURBO DNA-free kit (Invitrogen by Thermo Fischer Scientific). Template cDNA was synthesized using the GoTaq 2-Step RT-qPCR System using 0.5 ng of RNA as a standard starting concentration. RNA starting concentrations were incrementally increased in an effort to detect low-copy number transcripts. All quantitative PCR (qPCR) was done following the manufacturer’s protocols. The *rrsA* gene was used as a reference gene and amplified using the rrsA_rt_F/rrsA_rt_R primers (Table 1) (39). The *gox1969* and *bamB* genes were amplified using the gox1969_rt_F/gox1969_rt_R and bamB_rt_F/bamB_rt_R primers, respectively (Table 1). Fold changes in expression were calculated using the Plaffl method (44).

### Novobiocin tolerance assay

Overnight cultures of *E. coli* K12 pASK-IBA3, *E. coli* ∆*bamB* pASK-IBA3, and *E. coli* ∆*bamB* pGox1969 were diluted to approximately 0.1 OD_600_, and the pGox1969 plasmid was induced by the addition of ATc to a final concentration of 25 ng/mL. Cultures were induced at 24°C with 250-rpm shaking. At 4 h post induction, cultures were normalized to an OD_600_ of 0.1 in LB with various concentrations of novobiocin, and 200 µL distributed onto a 96-well plate. Growth was monitored using a SpectraMax M3 plate reader (Molecular Devices) at 600 nm, 24°C, with shaking in between reads. Growth assays were done with at least three biological replicates each with three technical replicates. Doubling times were determined using RStudio and the Growthcurver package (32, 43).

### Data processing and statistical analysis

RStudio and Excel were used to perform all statistical analyses and generate all plots (Script 1). The R packages used in this study were dplyr, ggplot2, Growthcurver, multcomp, reshape2, and devtools (32, 45–49).

## SUPPLEMENTAL MATERIAL

Supplemental material spectrum.01060-24-s0001.pdf Fig. S1 and S2; Script S1.

ASM does not own the copyrights to Supplemental Material that may be linked to, or accessed through, an article. The authors have granted ASM a non-exclusive, world-wide license to publish the Supplemental Material files. Please contact the corresponding author directly for reuse.
