# Supplementary material for: Determining the functional role of the Gluconobacter oxydans GOX1969 protein as a BamB homolog
Source: Microbiol Spectr. 2024 Jun 25;12(8):e01060-24. doi: 10.1128/spectrum.01060-24 (PMC11302035; doi:10.1128/spectrum.01060-24)
Supplement: Supplemental material — Fig. S1 and S2; Script S1. [file spectrum.01060-24-s0001.pdf]

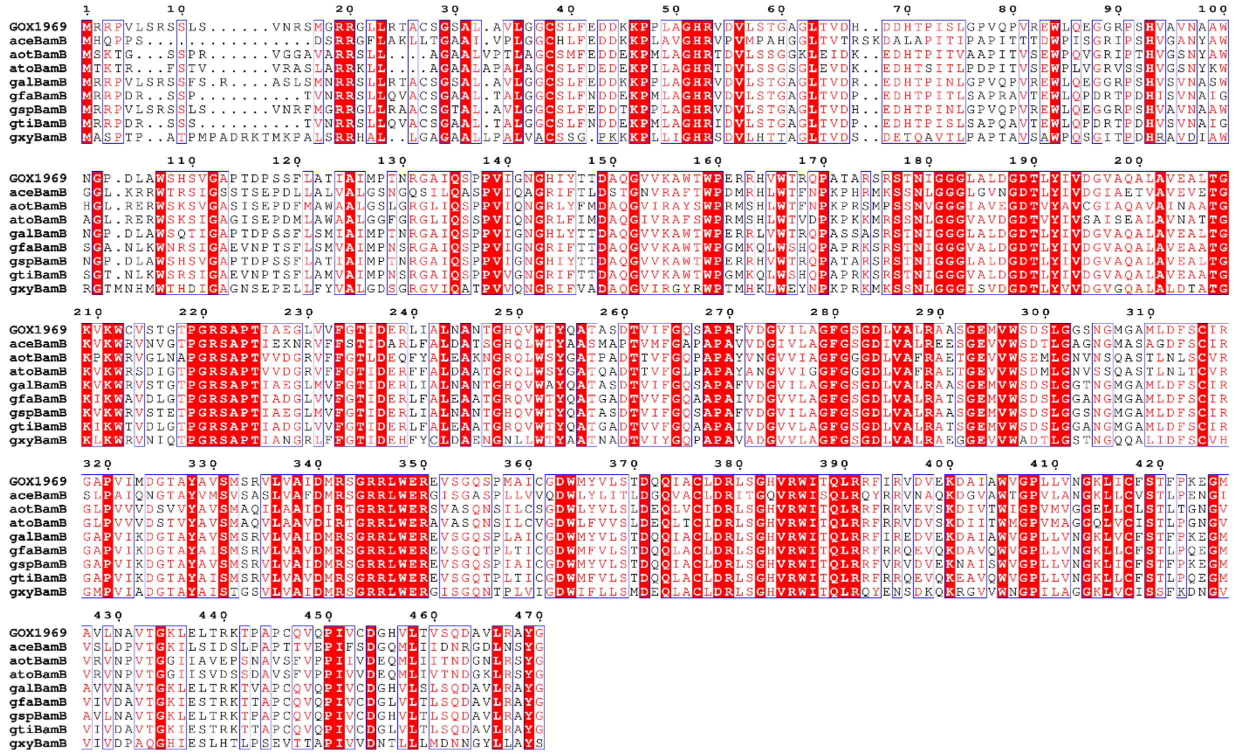


**Supplementary Figure 1. Predicted acetic acid bacteria BamB proteins have a high degree of sequence similarity with GOX1969.** Multiple sequence alignment of *G. oxydans* GOX1969 with BamB genes from *Acetobacter aceti* (UniProt: A0A1U9KF27), *Acetobacter orientalis* (UniProt: A0A2Z5ZEY2), *Acetobacter tropicalis* (UniProt: A0A291PKT9), *Gluconobacter albidus* (UniProt: A0A1U9KWN9), *Gluconobacter fraturii* (NCBI-ProteinID: UMM07592), *Gluconobacter sphaericus* (NCBI-ProteinID: QQX90715), *Gluconobacter xylinus* (UniProt: G2I4K6), *and Gluconobacter thailandicus* (UniProt: A0A149SPC9) produced using T-Coffee in Snapgene (39). Sequences are annotated for percent similarity using ESPript v.3.0 (40). Red boxes indicate strict sequence identity and red letters indicate sequence similarity. Blue frames indicate regions of similarity between the sequences.

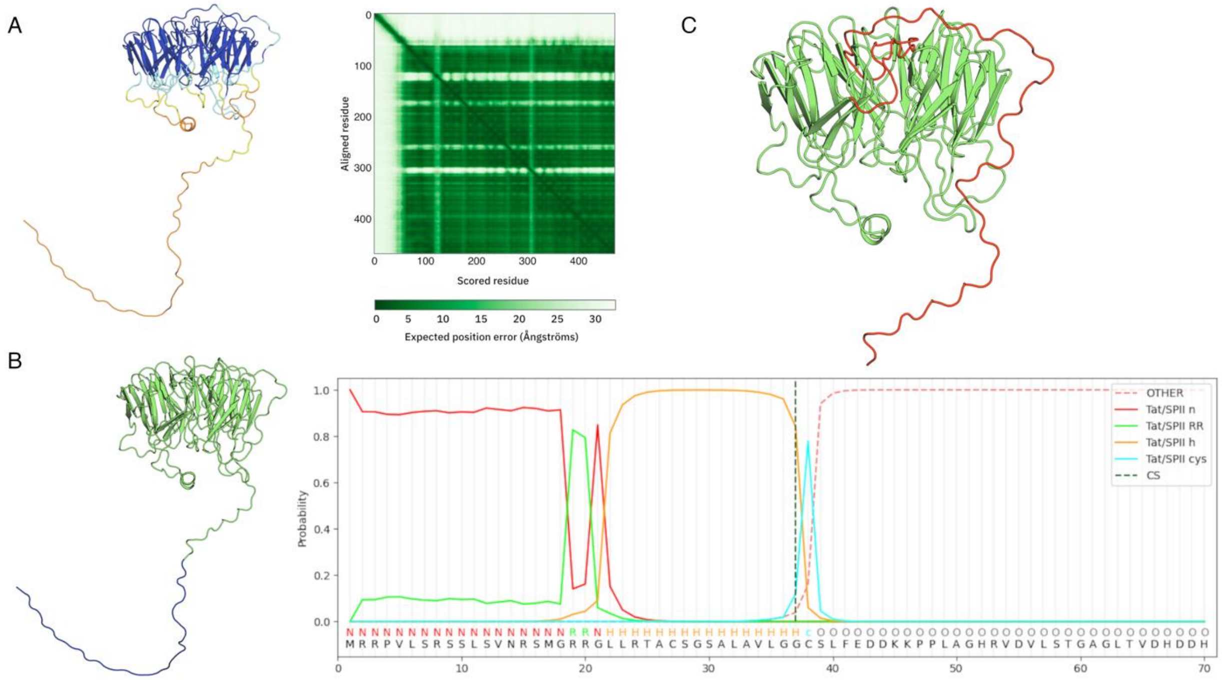


**Supplementary Figure 2. Refinement of the GOX1969 predicted model.** (A) Alphafold2 model of GOX1969 colored to show per-residue confidence scores (pLDDT) and its accompanying predicted alignment error plot. Dark blue coloring denotes pLDDT > 90, light blue coloring denotes a pLDDT > 70, yellow coloring denotes a pLDDT > 50, and orange coloring denotes a pLDDT < 50. (B) Alphafold2 model of GOX1969 colored to depict the SignalP6.0 predicted TAT/SPII signal peptide (shown in blue) and the SignalP-6.0 output plot. (C) N-Terminal obscuring loop shown on the GOX1969 predicted model in red.

**Script 1**

```
library("dplyr")
library("ggplot2")
library("growthcurver")
library("multcomp")
library("reshape2")
library("devtools")

cbPalette <- c("#E69F00", "#56B4E9", "#009E73", "#F0E442","#CC79A7","#E69F00", "#56B4E9", "#009E73",
"#F0E442","#CC79A7","#E69F00", "#56B4E9", "#009E73", "#F0E442","#CC79A7" )
palette(cbPalette)

ODreads <- read.csv("ODReads.csv")
platemap <- read.csv("platemap.csv")

shape_raw <- melt(ODreads, id=c("Time"), variable.name = "Well", value.name = "ODRaw600")
annotatedODraw <- inner_join(shape_raw, platemap, by="Well")
write.csv(annotatedODraw, "merged_data_raw.csv")

conf_int95_raw <- function(data) {
  n <- length(data)
  error <- qt(0.975, df=n-1) * sd(data)/sqrt(n)
  return(error)}

stats_raw <- annotatedODraw %>% group_by(Sample, Time) %>% summarize(N=length(ODRaw600),
Average=mean(ODRaw600),
CI95_raw=conf_int95_raw(ODRaw600)) %>%filter(!is.na(Sample))

stats_raw$Sample <-factor(stats_raw$Sample, levels = c("a","b","c","d"),
                    ordered = TRUE, labels = c(expression(paste(italic("E.coli "),"K12 pASK-IBA3 ATc")),
                                    expression(paste(italic("E.coli "),Delta,italic(bamB)," pASK-IBA3 ATc")),
                                    expression(paste(italic("E.coli "),Delta,italic(bamB)," pGox1969")),
                                    expression(paste(italic("E. coli "),Delta,italic(bamB)," pGox1969 ATc"))))

growth.curve <-ggplot(data=stats_raw, aes(x = Time, y = Average, color=Sample)) +
  theme_classic() +
  geom_point(aes(color=Sample), show.legend = T, size = 1.5) +
  geom_line(aes(color=Sample), show.legend = FALSE, lwd = 1) +
  geom_ribbon(aes(ymin=Average-CI95_raw, ymax=Average+CI95_raw, fill=Sample),color=NA, alpha=0.2,
show.legend = FALSE) +
  labs(x="Time (hr)", y=expression(paste("Bacterial Growth (OD"^600,")")), color="Strain", fill="Strain") +
  scale_x_continuous(expand = c(0,0)) +
  scale_y_continuous(limits = c(0,1),
              breaks = seq(0,1, by = 0.2),
              expand = c(0,0)) +
  scale_color_manual(values=c("#E69F00", "#56B4E9", "#009E73", "#F0E442"),
              labels = c(expression(paste(italic("E.coli "),"K12 pASK-IBA3 ATc")),
                   expression(paste(italic("E.coli "),Delta,italic(bamB)," pASK-IBA3 ATc")),
                   expression(paste(italic("E.coli "),Delta,italic(bamB)," pGox1969")),
                   expression(paste(italic("E. coli "),Delta,italic(bamB)," pGox1969 ATc")))) +
  scale_fill_manual(values=c("#E69F00", "#56B4E9", "#009E73", "#F0E442"),
             labels = c(expression(paste(italic("E.coli "),"K12 pASK-IBA3 ATc")),
```

```
                expression(paste(italic("E.coli "),Delta,italic(bamB)," pASK-IBA3 ATc")),
                expression(paste(italic("E.coli "),Delta,italic(bamB)," pGox1969")),
                expression(paste(italic("E. coli "),Delta,italic(bamB)," pGox1969 ATc")))) +
 theme(axis.title.x.bottom = element_text(size = 20),
    axis.title.y.left = element_text(size = 20),
    axis.text = element_text(size=13,color = "black"),
    legend.text.align = 0,
    legend.position = c(0.25,0.8),
    legend.title = element_text(size=15),
    legend.text = element_text(size = 15),
    plot.margin = margin(0.3, #top
               0.2, #right
               0.2, #bottom
               0.2, #left"

plate <- SummarizeGrowthByPlate(ODreads)
write.csv(plate, file = "Doubling_Time_GrowthCurver.csv")
Td <- read.csv("td.csv")
Tdmelt <- melt(Td)

anova <- aov(value~variable, data=Tdmelt)

tuk <- glht(anova, linfct = mcp(variable = "Tukey"))
summary(tuk)
cld(tuk)
td.boxplot <- Tdmelt %>%
 ggplot(aes(x=variable, y=value, fill=variable)) +
 theme_classic() +
 stat_summary(fun = median, show.legend=FALSE, geom="crossbar") +
 geom_boxplot(outlier.color = "white",
       outlier.shape = 16, outlier.size = 2,
       fill = "white", notch = FALSE, lwd = 0.75) +
 geom_jitter(show.legend=T, width=0.25, shape=21, size=3, color="black") +
 labs(x="Bacterial Strain", y="Doubling Time (min)", color="Strain", fill="Strain") +
 scale_color_manual(values=c("#E69F00", "#56B4E9", "#009E73", "#F0E442"),
          labels = c(expression(paste(italic("E.coli "),"K12 pASK-IBA3 ATc")),
                expression(paste(italic("E.coli "),Delta,italic(bamB)," pASK-IBA3 ATc")),
                expression(paste(italic("E.coli "),Delta,italic(bamB)," pGox1969")),
                expression(paste(italic("E. coli "),Delta,italic(bamB)," pGox1969 ATc")))) +
 scale_fill_manual(values=c("#E69F00", "#56B4E9", "#009E73", "#F0E442"),
          labels = c(expression(paste(italic("E.coli "),"K12 pASK-IBA3 ATc")),
                expression(paste(italic("E.coli "),Delta,italic(bamB)," pASK-IBA3 ATc")),
                expression(paste(italic("E.coli "),Delta,italic(bamB)," pGox1969")),
                expression(paste(italic("E. coli "),Delta,italic(bamB)," pGox1969 ATc")))) +
 theme(plot.caption = element_text(hjust = 0.5, size = 15),
    axis.title.x.bottom = element_text(size = 0),
    axis.title.y.left = element_text(size = 20),
    axis.text.x = element_text(color = "black", size = 0),
    axis.ticks.x = element_blank(),
    axis.text.y = element_text(color = "black", size = 13),
    legend.text.align = 0,
    legend.position = c(0.8,0.5),
    legend.title = element_text(size=15),
```

```
    legend.text = element_text(size = 15),
    plot.margin = margin(0.1, #top
                    1.5, #right
                    0.1, #bottom
                    0.1, #left
                    "cm")) +
geom_line(data=tibble(x=c(1,2), y=c(135,135)),
     aes(x=x, y=y),
     inherit.aes=FALSE) +
geom_text(data=tibble(x=1.5, y=135),
       aes(x=x, y=y, label = '*'),size = 8,
       inherit.aes=FALSE)
```
